# IgG4-related lung disease and seropositive undifferentiated arthritis in a patient initially presenting with monoarthritis: a case report

**DOI:** 10.1186/s12890-026-04565-9

**Published:** 2026-08-06

**Authors:** Wenwen Peng, Tiefeng Li, Lun Zhang, Hanguang Dai, Qianqian Zhong, Yilei Zhao

**Affiliations:** 1 Department of Radiology, Beilun Branch of the First Affiliated Hospital, School of Medicine, Zhejiang University, Ningbo, 315800 China; 2 Department of Radiology, The First Affiliated Hospital, School of Medicine, Zhejiang University, Hangzhou, 310003 China

**Keywords:** IgG4-related lung disease, Seropositive undifferentiated arthritis, Multidisciplinary team, Case report

## Abstract

**Background:**

IgG4-related lung disease (IgG4-RLD) is a fibroinflammatory pulmonary manifestation of IgG4-related disease (IgG4-RD). Its comorbidity with seropositive undifferentiated arthritis (SUA) is rare and may be overlooked due to overlapping clinical and immunological features.

**Case presentation:**

A 71-year-old man with acute left knee pain secondary to a meniscal tear with synovitis, as confirmed by MRI, was scheduled for arthroscopy. A preoperative chest radiograph incidentally revealed diffuse right lung lesions.

**Diagnostic workup and therapeutic strategy:**

Laboratory studies showed elevated rheumatoid factor (RF) and anti-cyclic citrullinated peptide (anti-CCP) antibodies. The first multidisciplinary team (MDT) discussion established a diagnosis of SUA and initiated disease-modifying antirheumatic drug (DMARD) therapy, which resolved the arthralgia but did not improve the lung lesions. A subsequent MDT review, incorporating chest CT findings (thickened bronchovascular bundles and consolidation) and lung biopsy results, confirmed the diagnosis of IgG4-RLD. Treatment was then adjusted to high-dose glucocorticoids combined with DMARDs. Both pulmonary and articular manifestations showed significant remission.

**Conclusion:**

This rare case of IgG4-RLD complicating SUA highlights the importance of considering IgG4-RLD in patients with seropositive inflammatory arthritis presenting with atypical pulmonary imaging findings, particularly when lung lesions fail to respond to conventional DMARD therapy. MDT collaboration is essential for accurate diagnosis and optimal management of these concomitant autoimmune conditions.

## Introduction

IgG4-related disease (IgG4-RD) is a systemic fibroinflammatory condition characterized by dense lymphoplasmacytic infiltration with abundant IgG4-positive plasma cells, often leading to tumefactive lesions in affected organs [1]. Pulmonary involvement, termed IgG4-related lung disease (IgG4-RLD), manifests as a spectrum of clinical and radiological features that can mimic interstitial lung disease, infection, or malignancy [2–4]. Seropositive undifferentiated arthritis (SUA) is defined as early inflammatory arthritis with RA-related autoantibody positivity that does not fulfill the 2010 American College of Rheumatology (ACR)/European League Against Rheumatism (EULAR) criteria for rheumatoid arthritis (RA) [5]. Notably, pulmonary involvement in RA-associated interstitial lung disease (RA-ILD) can occur even in the preclinical stage [6]. Here, we report a rare case of IgG4-RLD in a patient with SUA, and describe its imaging features and diagnostic process to raise awareness of such overlapping diseases.

### Case presentation

**Fig. 1:**
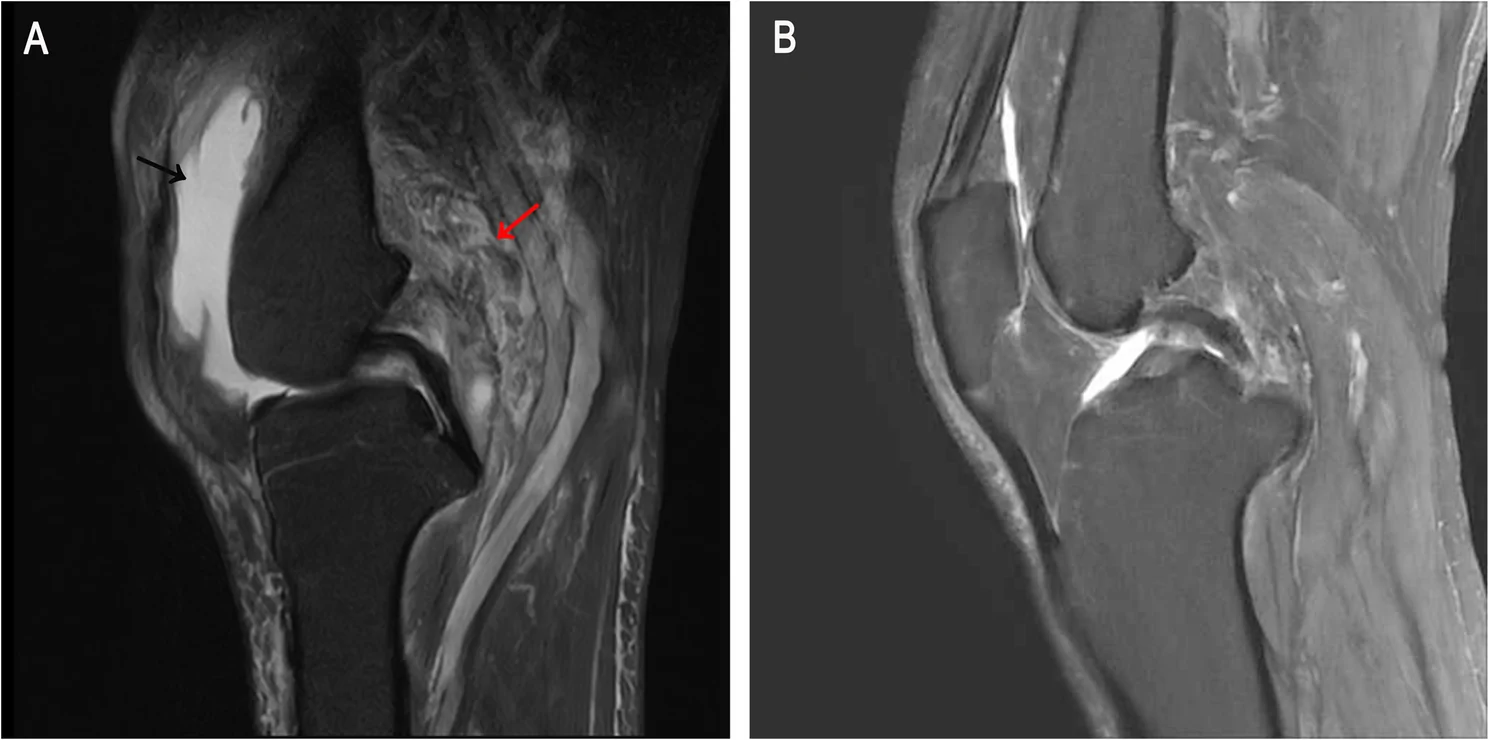
MRI findings of the left knee before and after combination therapy. **A **Left knee MRI (T2WI, sagittal; 2025.03.15) shows marked synovial thickening involving the posterior joint capsule, accompanied by a large joint effusion (→) with diffusely increased T2 signal intensity, consistent with active synovitis. **B **Left knee MRI (T2WI, sagittal; 2025.05.20) after DMARDs combined with high-dose glucocorticoid therapy shows substantial reduction in synovial thickening and near-complete resolution of the joint effusion

**Fig. 2:**
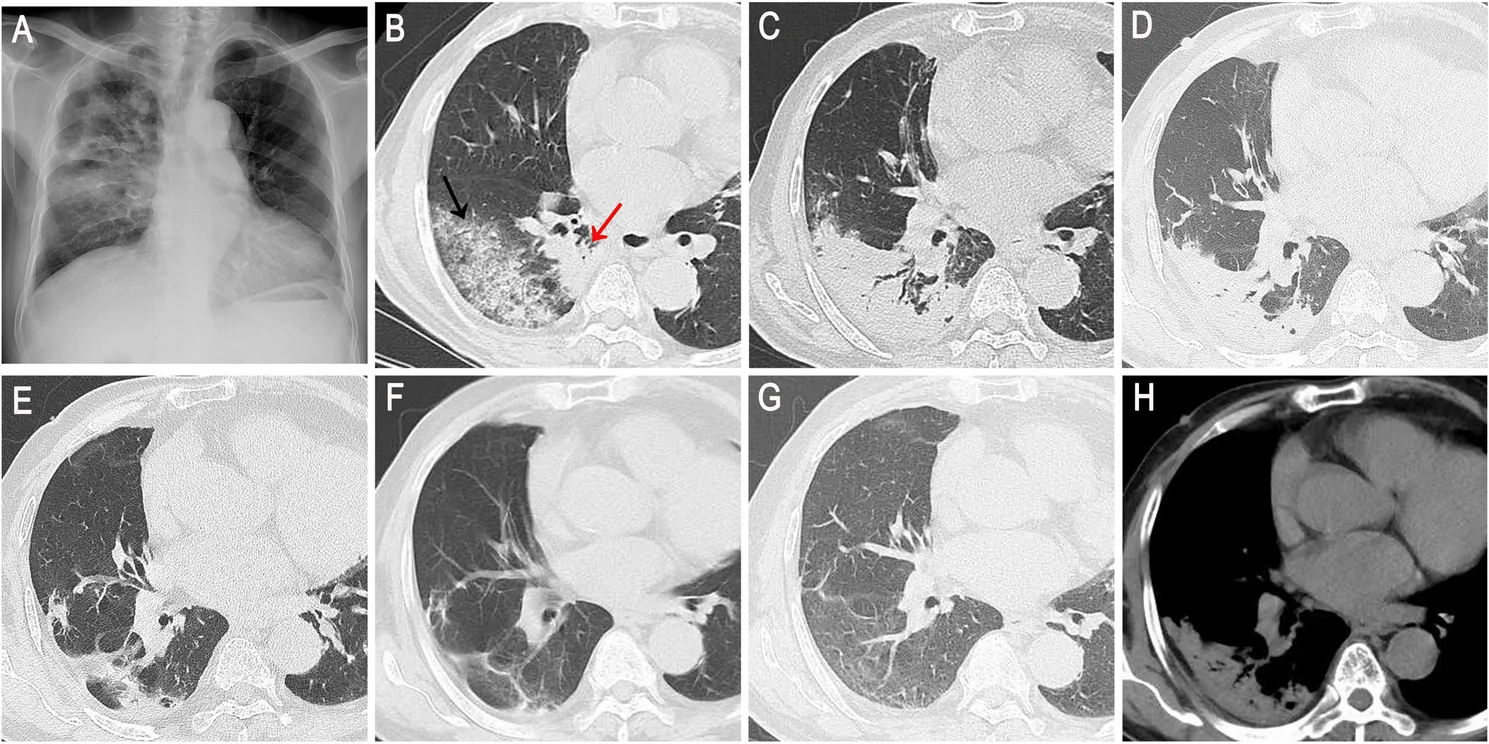
Imaging evolution of pulmonary lesions throughout the clinical course. **A **Chest X-ray (2025.03.16) shows diffuse opacities in the right lung. **B **Chest CT, lung window (2025.03.17) shows extensive ground-glass opacities (→), accompanied by thickening of the bronchovascular bundles and patchy areas of consolidation in the right lower lobe. **C **Follow-up chest CT after anti-infective therapy (2025.04.03) reveals progression to extensive consolidation. **D **Chest CT after DMARD therapy (2025.04.13) shows minimal improvement of consolidation in the right lung. **E **Chest CT after high-dose glucocorticoid combined with DMARD therapy (2025.04.26) demonstrates marked improvement. **F **Chest CT approximately one month later (2025.05.20) shows further resolution. **G **Three-month follow-up chest CT (2025.08.24) demonstrates stable residual linear and reticular opacities, without evidence of active inflammation. **H **Chest CT, mediastinal window (2025.04.13) shows thickening of the bronchovascular bundles and consolidation in the right lung, with no significant pleural effusion

A 71-year-old man presented to our hospital with acute pain and swelling in his left knee lasting one week. He had no significant past medical history and was taking no regular medications. Physical examination revealed prominent left knee swelling with limited range of motion. Knee MRI demonstrated a tear of the lateral meniscus with associated synovitis and joint effusion (Fig. 1A). Arthroscopic surgery was planned; however, a routine preoperative chest radiograph unexpectedly revealed diffuse opacities in the right lung (Fig. 2A), prompting further evaluation. Subsequent chest CT showed extensive ground-glass opacities, bronchovascular bundle thickening, and areas of consolidation in the right lung (Fig. 2B). These findings were initially diagnosed as interstitial lung disease with a possible superimposed infection.

The patient received a two‑week course of broad‑spectrum antibiotics; however, follow‑up CT showed progression of the pulmonary lesions (Fig. 2C). Bronchoscopy revealed copious viscous secretions in both main bronchi. Cultures and polymerase chain reaction tests of bronchoalveolar lavage fluid for common pathogens, including bacteria, fungi, mycobacteria, and viruses, were all negative. Laboratory tests showed markedly elevated inflammatory markers: erythrocyte sedimentation rate (ESR) 105 mm/h (reference range 0–20 mm/h) and C‑reactive protein (CRP) 119.9 mg/L (reference range < 10 mg/L). Rheumatoid factor (RF) was elevated at 717 IU/mL (reference range < 30 IU/mL), and anti‑cyclic citrullinated peptide (anti‑CCP) antibody was > 200 U/mL (reference range < 5 U/mL). Antinuclear antibodies (ANA), antineutrophil cytoplasmic antibodies (ANCA), and interferon‑gamma release assay for tuberculosis were negative. Serum IgG4 level was elevated to 2.14 g/L (reference range < 1.35 g/L).

**Fig. 3:**
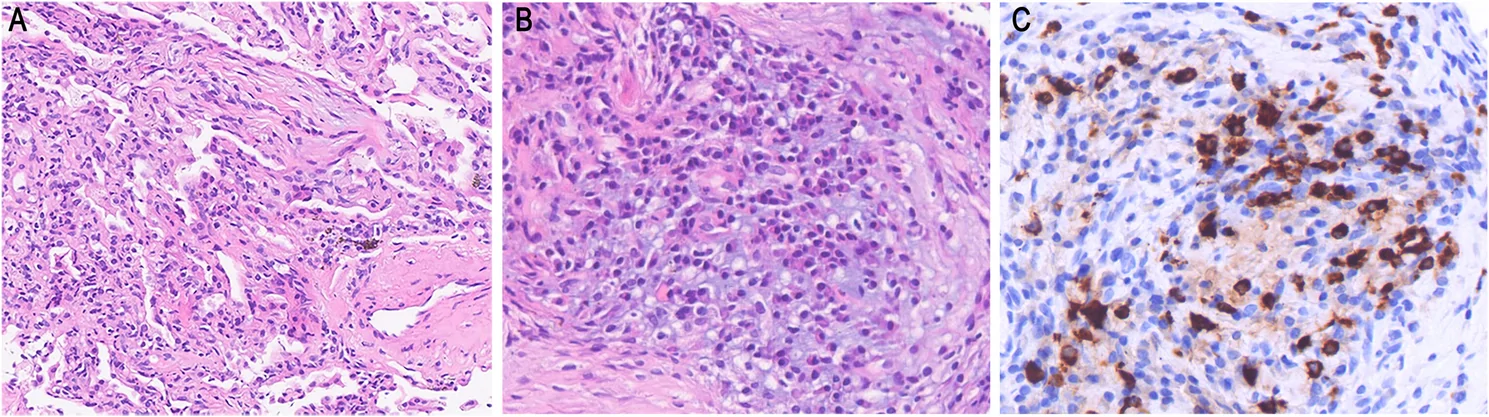
**A** Histopathological findings of the lung biopsy. **A **H&E staining (×200) shows diffuse mild interstitial fibrosis with lymphoplasmacytic infiltration. **B **H&E staining (×400) reveals dense plasma cell infiltration. **C **Immunohistochemical staining for IgG4 (×400) demonstrates numerous IgG4‑positive plasma cells (brown staining)

A multidisciplinary team (MDT) comprising rheumatologists, pulmonologists, and radiologists reviewed the case. Application of the 2010 ACR/EULAR classification criteria for rheumatoid arthritis (RA) yielded a total score of 4 (joint involvement: 1 large joint, 0 points; serology: high-positive RF and anti-CCP, 3 points; symptom duration > 6 weeks, 0 points; elevated acute-phase reactants, 1 point), which is below the threshold of 6 for classification as RA. The consensus was a diagnosis of seropositive undifferentiated arthritis (SUA). The pulmonary lesions were provisionally attributed to autoimmune-related interstitial lung disease, with RA-associated interstitial lung disease (RA-ILD) as the main differential diagnosis. Disease-modifying antirheumatic drug (DMARD) therapy consisting of sulfasalazine 4.5 g/d and leflunomide 10 mg/d, combined with prednisolone 10 mg/d was initiated. The left knee pain subsided within a week, but the lung lesions showed only minimal improvement (Fig. 2D). This discordance between articular and pulmonary responses prompted a second MDT review. Re-evaluation of the chest CT images highlighted features atypical for RA-ILD: predominant involvement of the right lung with bronchovascular bundle thickening and consolidation. IgG4-related lung disease (IgG4-RLD) was therefore suspected, and a CT-guided percutaneous lung biopsy was performed. Histopathological examination showed dense lymphoplasmacytic infiltration with interstitial fibrosis (Fig. 3A and B). Masson trichrome stain revealed focal areas suspicious for storiform fibrosis. Immunohistochemical staining revealed numerous IgG4-positive (IgG4+) plasma cells, with an IgG4+/IgG+ cell ratio > 40% and > 10 IgG4 + plasma cells per high-power field (Fig. 3C), meeting the histological criteria for IgG4-related disease (IgG4-RD). Abdominal CT revealed no extrapulmonary involvement.

**Fig. 4:**
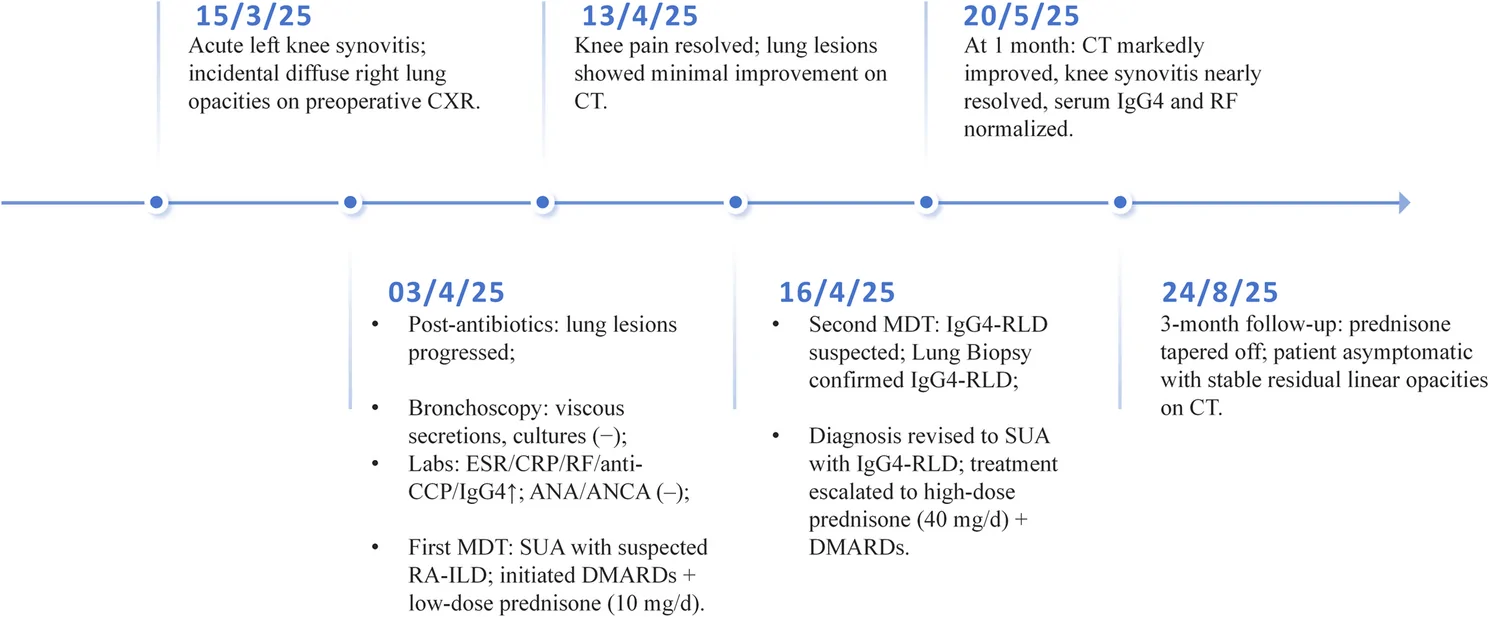
Timeline of the patient’s clinical course from symptom onset to follow-up

Based on these findings, a diagnosis of IgG4-RLD complicating SUA was established. As the initial 10 mg/day prednisone was selected for arthritic control and inadequate for lung involvement, the treatment regimen was intensified to high-dose glucocorticoids (prednisone 40 mg/day) combined with continued DMARD therapy. A follow-up chest CT two weeks later demonstrated marked regression of the pulmonary lesions (Fig. 2E). At one month, further improvement was noted (Fig. 2F), and the knee synovitis had nearly resolved (Fig. 1B). Serum IgG4 and RF levels normalized. Prednisone was gradually tapered over three months, and the patient remained asymptomatic with stable imaging findings at the most recent follow-up (Fig. 2G). The clinical course and timeline are illustrated in Fig. 4.

## Discussion

IgG4-RD is a systemic fibroinflammatory disorder, with approximately 20% of cases involving the lungs [7]. The pathogenesis remains unclear, and diagnosis requires a comprehensive assessment integrating clinical, radiological, serological, and histopathological findings. In the present case, the diagnostic process was complicated by the coexistence of two autoimmune conditions.

### Diagnostic considerations

This patient presented with acute monoarthritis, and imaging revealed a meniscal tear with synovitis. Despite the potential mechanical cause, the marked systemic inflammation and elevated RF and anti-CCP antibodies indicated an autoimmune aetiology rather than simple trauma. Monoarthritis is an atypical phenotype for RA-spectrum disease. Even elderly-onset RA (EORA) can manifest as large-joint polyarthritis [8], whereas this patient had isolated single-joint involvement without classic small-joint synovitis. Application of the 2010 ACR/EULAR criteria yielded a total score of 4 (serological and inflammatory indices), below the 6-point threshold for RA classification. While these criteria are primarily a research tool for standardizing clinical trial enrolment rather than an independent diagnostic instrument in routine practice, their standardized application helps identify patients at high risk of RA. Since the classification threshold was not met in this case, diagnosing early RA based solely on positive serological markers and atypical monoarticular involvement could carry a substantial risk of overdiagnosis. Furthermore, “pre-RA” is a retrospective concept defined by EULAR consensus, reserved for patients later confirmed to have RA, and is not applicable prospectively. In accordance with the EULAR-recommended terminology for RA, this patient presented with inflammatory arthritis after the exclusion of other known diseases; this condition meets the clinical definition of undifferentiated arthritis (UA) [9, 10]. In combination with the positive serological profiles, we established the clinical diagnosis as seropositive undifferentiated arthritis (SUA) [11].

IgG4-related synovitis was also considered. Although synovial biopsy was not performed and this diagnosis could not be entirely excluded, the rapid resolution of joint symptoms with DMARDs, in contrast to the minimal improvement in pulmonary lesions, argued against IgG4-RD as the cause of the arthritis. Considering the clinical presentation, serological indicators, and therapeutic responses, the most likely diagnosis was SUA. Starting DMARDs at this stage can control symptoms and may delay or even prevent progression to RA, thus improving long-term outcomes [12].

The pulmonary findings presented a greater diagnostic dilemma. When the initial antibiotic trial failed and bronchoscopic investigations were negative, RA-ILD became the leading consideration, given the newly established SUA diagnosis. However, RA-ILD typically manifests as usual interstitial pneumonia (UIP) or nonspecific interstitial pneumonia (NSIP) patterns, with subpleural and basal-predominant reticulation, traction bronchiectasis, and honeycombing [13]. In contrast, this patient exhibited predominantly unilateral bronchovascular bundle thickening and consolidation, a pattern more characteristic of IgG4-RLD [14]. The diagnosis was ultimately confirmed by significantly elevated serum IgG4 levels, along with histological evidence of extensive IgG4 + plasma cell infiltration on a lung biopsy.

IgG4-RLD overlaps clinically and radiologically with several other diseases, posing a diagnostic challenge. Given the unilateral consolidation observed in this case, the differential workup should encompass organizing pneumonia (OP), inflammatory pseudotumor (IPT), lymphoma, occult infection, and ANCA-negative vasculitis. Table 1 provides a comparative summary of their key clinical and radiological characteristics.

**Table 1:**
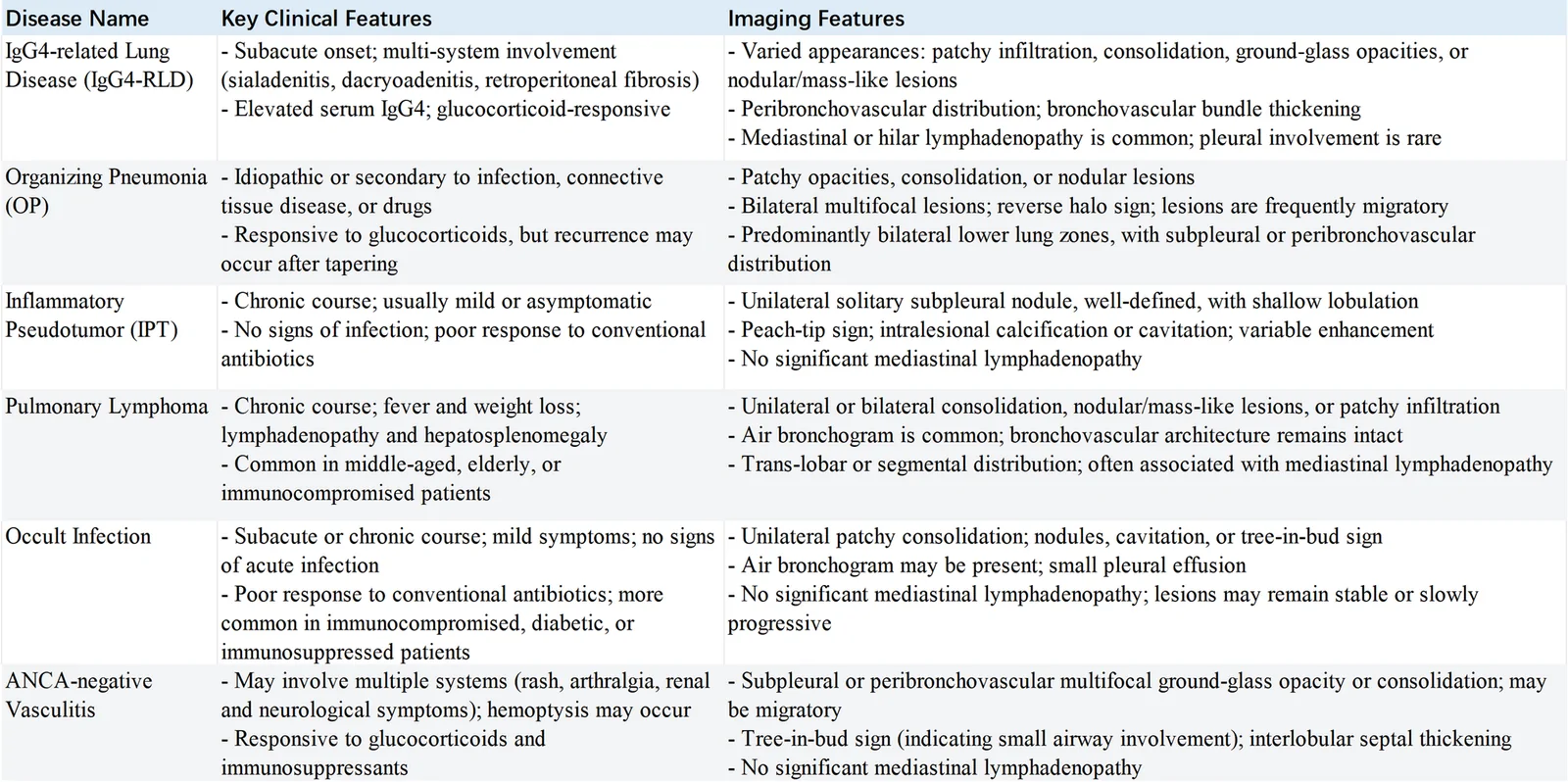
Differential diagnosis of unilateral pulmonary concolidation

### Evidence for coexistence and therapeutic implications

Evidence for the coexistence of IgG4-RLD and SUA is limited. However, well-established evidence confirms that IgG4-RD can develop in the context of RA. Large-scale retrospective studies indicate that approximately 8.5% of patients with IgG4-RD have a comorbid autoimmune disease, with RA accounting for 1.3% of cases [15–17]; isolated case reports have documented RA coexisting with IgG4-RD in the uterus, kidneys, and retroperitoneum [18–20]. Furthermore, studies have shown that 30.3% of patients with RA exhibit elevated serum IgG4 levels [21]. This may be attributed to chronic antigenic stimulation driving Th2-mediated immune responses and promoting the production of IgG4-class autoantibodies [22].

The optimal management for IgG4-RLD concurrent with SUA remains undefined. IgG4-RD typically responds well to glucocorticoids. In this case, low-dose prednisone combined with DMARD therapy adequately controlled articular symptoms but was insufficient for pulmonary lesions, necessitating escalation to high-dose glucocorticoids. The excellent response to combined therapy aligns with previous reports of IgG4-RD with comorbid autoimmune diseases [15], providing clinical support for combined therapy in such overlapping conditions.

### Limitations

Several limitations merit acknowledgment. First, synovial biopsy was not performed, which precluded definitive characterization of the knee joint pathology. Second, while pathological examination revealed classic IgG4-RD features with extensive infiltration of IgG4 + plasma cells and multiple foci of suspected storiform fibrosis, obliterative phlebitis was not clearly identified in the available specimens, constituting a diagnostic limitation of this case. Third, while abdominal CT revealed no extrapulmonary involvement, more sensitive imaging such as ¹⁸F-FDG PET/CT might have detected subclinical disease.

## Conclusion

This rare case of IgG4-RLD occurring in a patient with SUA highlights the importance of considering IgG4-RLD in patients with SUA who present with atypical imaging findings, particularly when pulmonary lesions progress despite adequate control of articular symptoms. Close collaboration among radiologists, rheumatologists, and pulmonologists is essential for timely and accurate diagnosis. High-dose glucocorticoids combined with DMARD therapy can achieve favorable outcomes.

## Data Availability

Data available from the corresponding author upon request.

## Abbreviations

IgG4-RD: IgG4-Related Disease
IgG4-RLD: IgG4-Related Lung Disease
SUA: Seropositive Undifferentiated Arthritis
RA: Rheumatoid Arthritis
RA-ILD: RA-associated interstitial lung disease
DMARD: Disease-Modifying Antirheumatic Drug
RF: Rheumatoid Factor
ANCA: Antineutrophil Cytoplasmic Antibodies
ANA: Antinuclear Antibodies
ESR: Erythrocyte Sedimentation Rate
MDT: Multidisciplinary Team
EORA: Elderly-Onset Rheumatoid Arthritis
UIP: Usual Interstitial Pneumonia
NSIP: Nonspecific Interstitial Pneumonia

## References

[1] Li PH, Ko KL, Ho CT, Lau LL, Tsang RK, Cheung TT, Leung WK, Lau CS. Immunoglobulin G4-related disease in Hong Kong: clinical features, treatment practices, and its association with multisystem disease. Hong Kong Med J. 2017;23(5):446–53. 10.12809/hkmj176229 28862143

[2] Zheng X, Zou W, Zou S, Ye J, Bao Z, Hong J, Song Y. IgG4-related Lung Disease: A Case Report and Review of the Literature. Altern Ther Health Med. 2023;29(8):320–3. 37632955

[3] Park DY, Kim SY, Bae SH, Lee JY. Immunoglobulin G4-Related Lung Disease Mimicking Lung Cancer: Two Case Reports. J Korean Soc Radiol. 2022;83(5):1168–74. 10.3348/jksr.2021.0158 PMC9574289 36276215

[4] Shafiq I, Ghorab O, Abuzakouk M, Mohammed S, Uzbeck MH, Wahla AS. IgG4-related lung disease mimicking lung cancer. Radiol Case Rep. 2022;17(3):931–4. 10.1016/j.radcr.2021.12.060 PMC8777243 35079315

[5] Aletaha D, Neogi T, Silman AJ, Funovits J, Felson DT, Bingham CO 3rd, Birnbaum NS, Burmester GR, Bykerk VP, Cohen MD, et al. 2010 rheumatoid arthritis classification criteria: an American College of Rheumatology/European League Against Rheumatism collaborative initiative. Ann Rheum Dis. 2010;69(9):1580–8. 10.1136/ard.2010.138461 20699241

[6] McDermott GC, Doyle TJ, Sparks JA. Interstitial lung disease throughout the rheumatoid arthritis disease course. Curr Opin Rheumatol. 2021;33(3):284–91. 10.1097/BOR.0000000000000787 PMC8268047 33625044

[7] Moura MC, Gripaldo R, Baqir M, Ryu JH. Thoracic Involvement in IgG4-Related Disease. Semin Respir Crit Care Med. 2020;41(2):202–13. 10.1055/s-0039-1700995 32279291

[8] Pavlov-Dolijanovic S, Bogojevic M, Nozica-Radulovic T, Radunovic G, Mujovic N. Elderly-onset rheumatoid arthritis: characteristics and treatment options. Med (Kaunas). 2023;59(10):1878. 10.3390/medicina59101878 PMC10608066 37893596

[9] Gerlag DM, Raza K, van Baarsen LG, Brouwer E, Buckley CD, Burmester GR, Gabay C, Catrina AI, Cope AP, Cornelis F, et al. EULAR recommendations for terminology and research in individuals at risk of rheumatoid arthritis: report from the Study Group for Risk Factors for Rheumatoid Arthritis. Ann Rheum Dis. 2012;71(5):638–41. 10.1136/annrheumdis-2011-200990 PMC3329228 22387728

[10] van der Helm AHM, Aletaha D. How to treat undifferentiated arthritis today or tomorrow? A consideration of treatment recommendations in light of current evidence. Ann Rheum Dis. 2025;84(4):521–8. 10.1016/j.ard.2025.01.036 39956701

[11] Niu HQ, Yuan C, Yan C, Li N, Lei YS, Li X, Ru J, Li XF. Decreased numbers and sex-based differences of circulating regulatory T cells in patients with seropositive undifferentiated arthritis. Ther Adv Chronic Dis. 2021;12:2040622320986721. 10.1177/2040622320986721 PMC7925950 33717426

[12] Combe B, Landewe R, Daien CI, Hua C, Aletaha D, Álvaro-Gracia JM, Bakkers M, Brodin N, Burmester GR, Codreanu C, et al. 2016 update of the EULAR recommendations for the management of early arthritis. Ann Rheum Dis. 2017;76(6):948–59. 10.1136/annrheumdis-2016-210602 27979873

[13] Ebner L, Christodoulidis S, Stathopoulou T, Geiser T, Stalder O, Limacher A, Heverhagen JT, Mougiakakou SG, Christe A. Meta-analysis of the radiological and clinical features of Usual Interstitial Pneumonia (UIP) and Nonspecific Interstitial Pneumonia (NSIP). PLoS ONE. 2020;15(1):e0226084. 10.1371/journal.pone.0226084 PMC6957301 31929532

[14] Gallo JR, Froullet C, Varizat A, Calvo RA, Pisoni C, Cosatti M, Bertoli A, Paira S. Clinical and Imaging Pulmonary Manifestations in IgG4-Related Disease. J Clin Rheumatol. 2025;31(2):47–52. 10.1097/RHU.0000000000002160 39823236

[15] Yang X, Nie Y, Li J, Peng Y, Zhang J, Zhang N, et al. IgG4-related disease with comorbid autoimmune diseases: a retrospective study from a large cohort. Clin Rheumatol. 2025;44(12):4985–4994. 10.1007/s10067-025-07749-6 41123771

[16] El-Saadany H, El-Saadany H, Tharwat S, Soliman W, Shereef SE, Elhefny A, Yehia A, El-Shebini E, Khalil N, El-Hindawy A, et al. Discriminative features of immunoglobulin G4-related disease (IgG4-RD) and associated autoimmune rheumatic diseases (ARDs) in a nationwide observational cohort: study from the Egyptian College of Rheumatology. Clin Rheumatol. 2025;44(2):747–56. 10.1007/s10067-024-07274-y PMC11775065 39751976

[17] Batani V, Lanzillotta M, Mahajne J, Pedica F, Palumbo D, Venturini E, Mapelli P, Bossi P, Dagna L, Della-Torre E. Association of IgG4-related disease and systemic rheumatic disorders. Eur J Intern Med. 2023;111:63–8. 10.1016/j.ejim.2023.03.015 36935250

[18] Ogawa T, Tsujimoto K, Shimagami H, Fukuda C, Hirano T. The Onset of IgG4-related Retroperitoneal Fibrosis under Administration of a TNF Inhibitor in a Rheumatoid Arthritis Patient. Intern Med. 2023;62(21):3251–4. 10.2169/internalmedicine.1326-22 PMC10686727 36927972

[19] Eroglu E, Sipahioglu MH, Senel S, Ertas SK, Savas S, Ozturk F, Kocyigit I, Tokgoz B, Oymak O. Successful treatment of tubulointerstitial nephritis in immunoglobulin G4-related disease with rituximab: A case report. World J Clin Cases. 2019;7(16):2309–15. 10.12998/wjcc.v7.i16.2309 PMC6718787 31531324

[20] Pacyna RR, Cipriani NA, Mathew MS, Kim JS. IgG4-related disease mimicking gynecologic malignancy. Gynecol Oncol Rep. 2023;45:101137. 10.1016/j.gore.2023.101137 PMC9879761 36714372

[21] Yu KH, Chan TM, Tsai PH, Chen CH, Chang PY. Diagnostic Performance of Serum IgG4 Levels in Patients With IgG4-Related Disease. Med (Baltim). 2015;94(41):e1707. 10.1097/MD.0000000000001707 PMC4616795 26469909

[22] Engelmann R, Brandt J, Eggert M, Karberg K, Krause A, Neeck G, Mueller-Hilke B. IgG1 and IgG4 are the predominant subclasses among auto-antibodies against two citrullinated antigens in RA. Rheumatology (Oxford). 2008;47(10):1489–92. 10.1093/rheumatology/ken336 18703528

